# Obstructive Sleep Apnea, Oxidative Stress, and Cardiovascular Disease: Evidence from Human Studies

**DOI:** 10.1155/2015/608438

**Published:** 2015-06-08

**Authors:** Hans-Joachim Eisele, Philipp Markart, Richard Schulz

**Affiliations:** Department of Pneumology, University Hospital of Marburg, Campus Fulda, Pacelliallee 4, 36043 Fulda, Germany

## Abstract

Obstructive sleep apnea (OSA) is a frequent disease mainly affecting obese people and caused by repetitive collapse of the upper airways during sleep. The increased morbidity and mortality of OSA are mainly thought to be the consequence of its adverse effects on cardiovascular (CV) health. In this context, oxidative stress induced by nocturnal intermittent hypoxia has been identified to play a major role. This is suggested by biomarker studies in OSA patients showing excessively generated reactive oxygen species from leukocytes, reduced plasma levels of nitrite and nitrate, increased lipid peroxidation, and reduced antioxidant capacity. Biopsy studies complement these findings by demonstrating reduced endothelial nitric oxide synthase expression and increased nitrotyrosine immunofluorescence in the vasculature of these patients. Furthermore, oxidative stress in OSA correlates with surrogate markers of CV disease such as endothelial function, intima-media thickness, and high blood pressure. Continuous positive airway pressure therapy reverses oxidative stress in OSA. The same may be true for antioxidants; however, more studies are needed to clarify this issue.

## 1. Introduction

Obstructive sleep apnea (OSA) is the most common form of sleep-disordered breathing (SDB) and represents a major public health problem. It is caused by repetitive collapse of a narrow upper airway during sleep [1]. The main risk factors of the disease are obesity, male gender, and advanced age [2] but it may also occur in lean subjects, women, and children. Earlier studies have reported that OSA affects 2% and 4% of middle-aged women and men, respectively [3]. However, owing to the global obesity epidemic, these features might even underestimate the actual prevalence of OSA as suggested by more recent data from epidemiological studies [4].

The episodes of OSA are terminated by arousals. These events disturb normal sleep architecture and can lead to excessive daytime sleepiness in many patients. Furthermore, the risk of traffic accidents is increased. Finally, OSA is linked to enhanced cardiovascular (CV) morbidity and mortality [5]. The gold standard in the diagnosis of OSA is polysomnography and the mainstay of its treatment is continuous positive airway pressure (CPAP) therapy. By creating a pneumatic splint, CPAP rapidly eliminates upper airway collapse and thereby also most sequelae of the disease [6].

The aim of the current review article is to summarize the evidence for oxidative stress being present in humans with OSA and its relation to CV disease. Of note, this has been paralleled by the results of animal studies evaluating the effects of intermittent hypoxia (IH) on the CV system of rodents. For further details, the interested reader is referred to an earlier article we have published in this journal [7].

## 2. Clinical Spectrum of OSA-Associated Cardiovascular Diseases

During the night, blood pressure (BP) surges can be observed in both the systemic and pulmonary circulation [8]. Similarly, cyclical variations of the heart rate emerge (i.e., sinus tachycardia/bradycardia [9]). Furthermore, cardiac rhythm disturbances may occur [10]. These include sinus arrests, atrioventricular conduction blocks, atrial fibrillation, and ventricular arrhythmias. It is hypothesized that through these arrhythmias OSA can cause sudden cardiac death; however, the evidence for this is still somewhat circumstantial [11].

The deleterious effects of OSA on the CV system are carried over into daytime hours. Thus, up to one half of these patients suffer from arterial hypertension. The OSA-associated arterial hypertension is characterized by a nondipping 24-hour BP pattern and a high percentage of refractory and masked hypertension [12–14]. Mainly through its pressor effects, OSA increases the risks for stroke, heart failure, and myocardial infarction [5]. In addition, this may lead to more rapid expansion of aortic aneurysms [15]. OSA can also cause pulmonary hypertension; however, this occurs in a lower proportion of patients and usually is not very pronounced [16]. Furthermore, it may be a risk factor for thromboembolic disorders such as deep vein thrombosis and pulmonary embolism [17]. Finally, otherwise healthy OSA patients can already display more subtle CV changes such as endothelial dysfunction and vascular remodeling [18, 19].

CV risk in OSA depends on the severity of SDB; that is, those patients with an apnea-hypopnea-index exceeding 30 per hour of sleep are mainly affected [5]. Most studies evaluating CV morbidity and mortality in OSA have primarily enrolled typical sleep laboratory cohorts, that is, middle-aged obese men.

However, more recent data show that CV risk is also increased in other subsets of OSA patients such as women and the elderly [20, 21]. Of note, this is independent from established CV risk factors such as advanced age, obesity, smoking, and metabolic disorders.

It is well known that sleep characteristics such as sleep duration and the ability to fall asleep or maintain normal sleep can impact on CV health [22, 23]. Consequently, those OSA patients who suffer from insomnia or excessive daytime sleepiness have a higher CV risk than those who do not [24].

## 3. Pathophysiology of OSA-Associated Cardiovascular Diseases

Over the last two decades, the intermediary pathways linking OSA to CV disease have been further elucidated. The main trigger of these metabolic and cellular changes is IH, that is, the nocturnal hypoxia-reoxygenation events accompanying OSA. Other OSA-associated stimuli such as hypercapnia, arousals, and intrathoracic pressure swings may also play significant roles; however, this has been less extensively studied.

One major pathogenic mechanism of CV disease in OSA is sympathetic activation [25]. It is mediated by enhanced chemoreflex activity in the carotid body. Furthermore, inflammatory changes within the vasculature and the adipose tissue which are due to NF*κ*B activation have been described [26, 27]. Additional pathways include the upregulation of hypoxia-sensitive endothelial-derived peptides such as endothelin and vascular endothelial growth factor, enhanced coagulation, and decreased fibrinolysis as well as disturbed vascular repair mechanisms [28–31]. Finally, OSA is linked to insulin resistance and dyslipidemia which may also contribute to the emergence of CV disease [32, 33].

Another potential mechanism of CV disease in OSA is an increased oxidative stress. Under physiologic conditions, there is a balance between aggressive and protective factors influencing redox control. The term “oxidative stress” refers to an imbalance between reactive oxygen species (ROS) and reactive nitrogen species production and the system of antioxidant defense resulting in a serious disorder of redox homeostasis [34]. Oxidative stress has been reported to underlie the process of aging and plays a role in the pathogenesis of such different diseases as cancer, chronic inflammatory, and neurodegenerative disorders [35]. Furthermore, there is a strong correlation between oxidative stress and CV diseases such as atherosclerosis, hypertension, and endothelial dysfunction [36].

## 4. Evidence for Oxidative Stress in Humans with OSA

**Figure 1:**
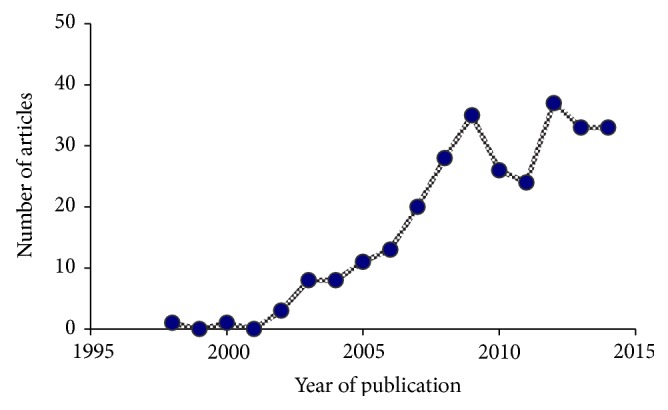
Publications found in* Pubmed* with the search terms of “sleep apnea,” “cardiovascular” and “oxidative stress.”

In the following paragraphs, the various aspects of oxidative stress in patients with OSA will be discussed in more detail. For this purpose, a* Pubmed* search was performed with the search terms “sleep apnea,” “cardiovascular” and “oxidative stress.” As can be seen from Figure 1 this search yielded a growing number of publications dating back to the late 1990s. Overall, a total of 292 entries were retrieved. Only full-length original publications dealing with human subjects with OSA were taken into consideration. With a few exceptions, replication studies as well as those not evaluating the effects of CPAP therapy on oxidative stress parameters were excluded. Following this strategy, 36 publications were selected for this review article.

### 4.1. Methodological Aspects of Studies

The studies exploring oxidative stress in OSA have employed different methodological approaches. Most commonly, biomarkers of oxidative stress were determined in peripheral venous blood samples obtained after sleep. Other studies aimed to integrate CV surrogate markers such as vasoreactivity and intima-media thickness (IMT). Finally, some investigators have applied more invasive procedures, that is, vascular biopsies.

Most studies enrolled patients with OSA and controls without SDB and measured the aforementioned parameters before and after CPAP therapy. The majority of these studies had low patient numbers. Furthermore, most of them were not randomized and uncontrolled; that is, they did not have a treatment arm with, for example, sham CPAP (i.e., with subtherapeutical pressure). Finally, many earlier studies recruited patients with comorbidities which might cause oxidative stress by itself. Therefore, it is recommended to restrict study participation to otherwise healthy, nonobese, and nonsmoking OSA patients when performing investigations in this field.

It should be acknowledged that the studies evaluating oxidative stress in OSA have not yielded unequivocal results. This may be due to varying severities of IH in the patients studied (i.e., frequency and extent of nocturnal oxygen desaturations [37–39]). Patient characteristics as age, gender, and body weight may also have an influence; however, this has not yet been substantiated. Furthermore, as mentioned above discrepancies in comorbidities may introduce some bias into analyses of oxidative stress parameters in these patients. Finally, differences in study design may play important roles in this context (i.e., full-night polysomnography versus split-night protocols [40]).

### 4.2. Enhanced Release of Superoxide from Leukocytes

The first studies demonstrating the presence of oxidative stress in humans with OSA were performed in our laboratory at the University of Giessen Lung Center, Germany, and at the University of Haifa, Israel, by the group of Lena and Peretz Lavie [41, 42]. It was observed that circulating neutrophils and monocytes of OSA patients exhibited markedly enhanced* in vitro* release of superoxide radical anions. This was the case in response to different stimuli such as formylmethionylleucylphenylalanine and phorbol myristate acetate but also in the unstimulated state. The increased oxidative burst of leukocytes in OSA is due to an activation of NADPH oxidase (NOX). The expression of this major superoxide-generating enzyme is upregulated in leukocytes from OSA patients. This was shown by measuring p22phox mRNA in peripheral blood mononuclear cells [43]. Furthermore, the serum levels of soluble NOX2-derived peptide, a marker of NOX activation by blood cells, decrease after CPAP therapy in patients with OSA [44].

### 4.3. Reduced Bioavailability of Nitric Oxide

Nitric oxide (NO) is synthesized from the amino acid L-arginine by the action of NO synthases (NOS). They occur in three different isoforms: endothelial, inducible, and neuronal. Endothelial NOS (eNOS) is the enzyme responsible for vascular NO production [45]. Importantly, some naturally occurring compounds can interfere with NO formation. For example, asymmetric dimethylarginine (ADMA) inhibits eNOS and arginase metabolizes L-arginine to L-ornithine and urea [46].

NO is the main endothelial-derived vasodilator. This effect is mediated by its second messenger guanosine 3′,5′-cyclic monophosphate [47]. NO also possesses other vasoprotective properties such as inhibition of platelet aggregation and adhesion molecule expression. The consequences of NO deficiency are, for example, elevated BP and enhanced atherosclerosis [48].

Through various pathways, oxidative stress can reduce NO bioavailability. First, superoxide directly interacts with NO resulting in the formation of peroxynitrite [49]. Second, the degradation of the eNOS cofactor tetrahydrobiopterin is accelerated [50]. Third, the activity of the enzyme dimethylarginine dimethylaminohydrolase is decreased, leading to higher levels of ADMA [51].

Due to its very short half-life of only a few seconds, it is challenging to measure NO itself. More feasible is the determination of its oxidation products nitrite and nitrate. In patients with OSA it was shown that serum levels of nitrite and nitrate are reduced when compared with controls without SDB [52–54]. The decreased nitrite and nitrate pool in OSA is probably due to decreased NO biosynthesis. This is suggested by the findings of increased plasma concentrations of ADMA and arginase in these patients [55–57].

### 4.4. Increased Oxidation of Lipids, Proteins, and DNA

Excessively generated ROS may lead to increased oxidation of biological compounds such as lipids, proteins, and DNA. Thus, OSA patients show evidence for increased lipid peroxidation as mirrored by elevated plasma concentrations of thiobarbituric acid reactive substances [58, 59]. In addition to inflammatory changes in response to IH, this may be responsible for accelerated atherosclerosis in affected patients.

Moreover, increased concentrations of 8-isoprostane can be found in exhaled breath condensate of OSA patients [60]. The isoprostanes are prostaglandin-like compounds formed* in vivo* from the free radical-catalyzed peroxidation of essential fatty acids (primarily arachidonic acid [61]). They enhance vasoconstrictor tone and may thus contribute to the development of arterial hypertension in OSA.

Some authors have reported on higher plasma advanced oxidation protein products in OSA versus non-OSA patients [62, 63]. DNA oxidation also takes place in OSA as suggested by the finding of increased urinary excretion of 8-hydroxy-2′-deoxyguanosine in patients with severe versus mild-to-moderate OSA [64]. In addition, the levels of DNA damage in peripheral blood lymphocytes as assessed by the cytokinesis-blocked micronucleus assay are increased in OSA [65]. Finally, a significant decrease in mitochondrial DNA copy number was observed in genomic DNA isolated from whole blood of OSA patients [66]. The pathophysiological significance of these changes in the context of OSA-associated CV disease awaits to be determined.

### 4.5. Reduced Antioxidant Capacity

The effects of ROS may be counterbalanced by antioxidant substances such as glutathione and vitamins A, C, and E. Some studies suggest that this defense line against oxidative stress is impaired in untreated patients with OSA. Antioxidant capacity as measured by the trolox equivalent antioxidant capacity assay was found to be reduced in OSA [67]. Furthermore, plasma total antioxidant status and levels of vitamins A and E were lower in OSA patients versus controls [68]. Finally, the antioxidant properties of serum albumin were shown to be impaired in OSA [69].

Oxidative stress in OSA may also stem from reduced activity of antioxidant enzymes. Thus, lower plasma levels of superoxide dismutase (SOD) have been described in OSA versus non-OSA patients [43, 70]. Similarly, a study evaluating microarray measures of gene transcript levels noted changes in genes encoding for antioxidant enzymes, that is, SOD and catalase [71]. Finally, OSA patients exhibit a decrease in paraoxonase-1, that is, an enzyme which protects lipoproteins from oxidation and thereby exerts antiatherogenic effects [59].

### 4.6. Vascular Biopsy Studies

Up to now, only a few studies have directly evaluated oxidative stress in the vasculature of OSA patients. This may be due to the reluctance of patients to have biopsies performed and also to the limited availability of these techniques. Jelic et al. harvested endothelial cells by introducing vascular guidewires into forearm veins. When compared to controls without SDB, the cells obtained from OSA patients showed reduced expression of eNOS and increased nitrotyrosine immunofluorescence [72]. Quite similar findings were later reported by Patt et al. who investigated arterioles isolated from forearm subcutaneous biopsies [73]. In a most recent study, endothelial tissue was obtained from gluteal biopsies of OSA and control subjects and eNOS uncoupling was identified as a novel pathway of OSA-associated endothelial dysfunction [74].

### 4.7. Correlation of Oxidative Stress in OSA with Surrogate Markers of CV Disease

OSA patients may exhibit endothelial dysfunction, that is, a reduction of endothelial-dependent vasorelaxation. This has been shown by various techniques investigating vasoreactivity as, for example, venous occlusion plethysmography and measurements of flow-mediated vasodilation (FMD) of the brachial artery [18, 75]. Endothelial dysfunction is a precursor lesion for both atherosclerosis and arterial hypertension and can already be detected in OSA patients without clinically overt CV disease.

Since endothelial-dependent vasodilation is mainly the result of NO release, it is not surprising that OSA patients with lower circulating levels of nitrite and nitrate have more severely impaired FMD [76]. Furthermore, the expression of eNOS correlates with FMD in these patients [72]. Finally, inverse relationships between %FMD and circulating levels of ADMA, soluble NOX2-derived peptide, and isoprostanes have been found [76, 77]. Taken together, these data clearly show that oxidative stress mechanisms underlie endothelial dysfunction in OSA.

Ultrasonographic studies have shown that OSA patients have greater IMT of their common carotid arteries when compared with non-OSA control subjects and that this is related to the degree of nocturnal hypoxia [19]. IMT reflects the early stages of atherosclerosis and predicts the risks of future stroke and myocardial infarction. Up to now, there is only one study which has investigated the correlation between oxidative stress and IMT in OSA. Monneret et al. found that the IMT of these patients was greater with higher urinary levels of isoprostanes [78].

Likewise, there is a paucity of data on the relationship between oxidative stress and BP in patients with OSA. Ip et al. have reported that the serum levels of nitrite and nitrate are inversely correlated with BP values in OSA [53]. Insofar, the NO deficiency characteristic of OSA may constitute one pathophysiological pathway for the development of arterial hypertension in these patients; however, it is felt that more investigations are required to support this hypothesis.

### 4.8. Effects of Therapeutical Interventions

As already stated, many of the studies evaluating the effects of CPAP therapy on oxidative stress in OSA had methodological drawbacks such as low patient numbers, inclusion of patients with comorbidities and observational design. Nevertheless, there is no doubt that CPAP therapy ameliorates or even eliminates oxidative stress in OSA. Thus, most changes in biomarker studies were rapidly reversible after only some nights of CPAP (Table 1). For instance, it was reported that CPAP therapy decreases the release of superoxide from leukocytes and increases circulating levels of nitrite and nitrate [41, 42, 52–54]. In addition, the immunohistochemical changes in endothelial cells consistent with oxidative stress were reversible after 1–3 months of CPAP therapy [72, 73]. These findings argue for more structural effects of CPAP therapy on the vasculature which may translate into a long-lasting suppression of oxidative stress in affected individuals.

On the other hand, it must be realized that a significant proportion of OSA patients is not able or willing to use CPAP therapy. Pilot studies suggest that antioxidants may be used to prevent or treat CV disease in such patients. We have shown that intravenous administration of the antioxidant vitamin C can acutely improve vasoreactivity in OSA [79]. This observation was later confirmed by another group [80]. Furthermore, it was found that the xanthine oxidase inhibitor allopurinol when given orally over two weeks increases FMD in OSA [81].

### 4.9. Prospects for Future Research

Despite the advancements highlighted in this review, we believe that more research is needed to ascertain a causal role of oxidative stress in OSA-associated CV disease. Appropriate studies should either investigate highly selected OSA patients (i.e., lean subjects without any comorbidities) or large cohorts allowing for statistical control of confounders. Furthermore, randomized controlled designs as, for example, with sham-CPAP treatment should be applied if possible.

One important area of future research certainly is the correlation of oxidative stress biomarkers in OSA with CV read-out parameters such as FMD, IMT, and BP. Long-term studies could possibly also evaluate the association with more robust CV endpoints such as the occurrence of myocardial infarction or stroke. This could, for instance, be accomplished by enrolling those patients who are noncompliant with CPAP therapy.

The same patient population may also help to establish the role of antioxidants in the treatment of OSA-associated CV diseases. In this context, it is an open question which antioxidants should be given and at what dose and route of administration. Regardless of these considerations, such studies would need larger patient numbers and long-term follow-up periods to delineate a therapeutic effect of antioxidants in OSA.

In addition to these more clinically oriented questions, research efforts should be undertaken to elucidate the basic mechanisms of oxidative stress in OSA. One possibility to accomplish this task is to go back from bench to bedside, that is, to see if the results of animal studies can be extrapolated to humans with the disease. In this context, numerous studies in mice and rats have shown that an upregulation of NOX underlies many of the CV sequelae of IH [7, 82]. Apart from NOX and its different isoforms, one could also look at other ROS-generating enzymes within the human body as, for example, xanthine oxidase, uncoupled eNOS, and mitochondrial respiratory chain enzymes. Finally, one should try to decipher the interaction of oxidative stress with other putative pathophysiological pathways of CV disease in OSA. In this context, animal studies suggest that ROS can mediate or aggravate sympathetic activation in response to IH [83]. Up to now, it is not known if this is also the case in patients with OSA.

## 5. Conclusions

**Figure 2:**
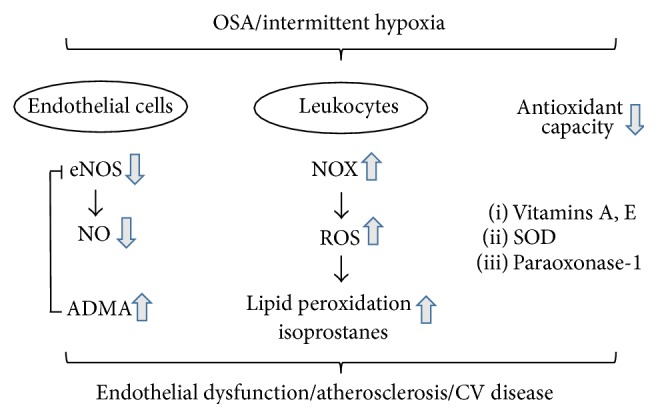
Oxidative stress as an intermediary pathway of OSA-associated CV disease. The intermittent hypoxia characteristic of OSA leads to increased oxidative burst of leukocytes via activation of NOX. Excessively produced ROS enhance lipid peroxidation and isoprostane formation. NO bioavailability is reduced by diminished expression of eNOS and its inhibition by ADMA. Finally, antioxidant capacity is impaired in affected patients. ADMA: asymmetric dimethylarginine; eNOS: endothelial nitric oxide synthase; NO: nitric oxide; NOX: NADPH oxidase; ROS: reactive oxygen species; SOD: superoxide dismutase.

In summary, there is accumulating evidence from human studies that untreated OSA causes oxidative stress and that effective CPAP therapy can reverse these abnormalities (Figure 2). Given the well-known role of ROS in the pathogenesis of CV disease, the oxidative stress induced by OSA may account for its associated CV morbidity and mortality; however, more studies are clearly needed in this area of research. In particular, the cardioprotective role of antioxidants in those patients not tolerating CPAP therapy and the basic mechanisms leading to oxidative stress in patients with OSA should be explored in more detail.

## Figures and Tables

**Table 1:** Overview of selected biomarker studies of oxidative stress in OSA.

Biomarker investigated	Author/year [reference number]	OSA/*n* = comorbidities yes/no	Controls/*n* = comorbidities yes/no	Change of biomarker	Effects of CPAP
PMN oxidative burst	Schulz et al./2000 [41]	18/yes	10/yes and 10/no	↑	↓
PMN oxidative burst	Dyugovskaya et al./2002 [42]	18/yes	31/yes	↑	↓
PMN p22phox mRNA	Liu et al./2009 [43]	107/yes	69/yes	↑	n.a.
Serum NOX2	Del Ben et al./2012 [44]	91/yes	47/yes	*↔*	↓
Nitrite and nitrate	Schulz et al./2000 [52]	21/yes	18/yes and 13/no	↓	↑
Nitrite and nitrate	Ip et al./2000 [53]	30/yes	40/no	↓	↑
Nitrite and nitrate	Alonso-Fernández et al./2009 [54]	31/no	15/no	↓	↑
ADMA	Barceló et al./2009 [56]	23/yes and 18/no	13/no	↑	n.a.
Arginase	Yüksel et al./2014 [57]	41/yes and 10/no	15/no	↑	n.a.
TBARS	Barceló et al./2000 [58]	14/yes	13/no	↑	↓
TBARS	Lavie et al./2004 [59]	59/yes and 55/no	30/yes	↑	↓
Exhaled 8-isoprostane	Carpagnano et al./2003 [60]	18/no	12/no	↑	↓
Urinary 8-OHdG	Yamauchi et al./2005 [64]	128/yes	n.a.	↑^*∗*^	n.a.
Vitamins A and E	Barceló et al./2006 [68]	47/yes	37/no	↓	*↔*
SOD	Wysocka et al./2008 [70]	41/no	39/no	↓	n.a.
Paraoxonase-1	Lavie et al./2004 [59]	59/yes and 55/no	30/yes	↓	n.a.

↑: increase, ↓: decrease, and *↔*: no change.

^*∗*^Patients with severe versus mild-to-moderate OSA.

ADMA: asymmetric dimethylarginine.

n.a. = not available.

NOX: NADPH oxidase.

8-OHdG: 8-hydroxy-2′-deoxyguanosine.

PMN: polymorphonuclear neutrophils.

SOD: superoxide dismutase.

TBARS: thiobarbituric acid reactive substances.

## Abbreviations

ADMA: Asymmetric dimethylarginine
BP: Blood pressure
CPAP: Continuous positive airway pressure
CV: Cardiovascular
eNOS: Endothelial nitric oxide synthase
FMD: Flow-mediated vasodilation
IH: Intermittent hypoxia
IMT: Intima-media thickness
NO: Nitric oxide
NOS: Nitric oxide synthase
NOX: NADPH oxidase
OSA: Obstructive sleep apnea
ROS: Reactive oxygen species
SDB: Sleep-disordered breathing
SOD: Superoxide dismutase.
